# Sensing coral reef connectivity pathways from space

**DOI:** 10.1038/s41598-017-08729-w

**Published:** 2017-08-24

**Authors:** Dionysios E. Raitsos, Robert J. W. Brewin, Peng Zhan, Denis Dreano, Yaswant Pradhan, Gerrit B. Nanninga, Ibrahim Hoteit

**Affiliations:** 1 Plymouth Marine Laboratory (PML), Plymouth, UK; 2 National Centre for Earth Observation, PML, Plymouth, UK; 3 King Abdullah University for Science and Technology (KAUST), Thuwal, Saudi Arabia; 4 Met Office, FitzRoy Road, Exeter, UK; 5 University of Cambridge, Cambridge, UK

## Abstract

Coral reefs rely on inter-habitat connectivity to maintain gene flow, biodiversity and ecosystem resilience. Coral reef communities of the Red Sea exhibit remarkable genetic homogeneity across most of the Arabian Peninsula coastline, with a genetic break towards the southern part of the basin. While previous studies have attributed these patterns to environmental heterogeneity, we hypothesize that they may also emerge as a result of dynamic circulation flow; yet, such linkages remain undemonstrated. Here, we integrate satellite-derived biophysical observations, particle dispersion model simulations, genetic population data and ship-borne *in situ* profiles to assess reef connectivity in the Red Sea. We simulated long-term (>20 yrs.) connectivity patterns driven by remotely-sensed sea surface height and evaluated results against estimates of genetic distance among populations of anemonefish, *Amphiprion bicinctus*, along the eastern Red Sea coastline. Predicted connectivity was remarkably consistent with genetic population data, demonstrating that circulation features (eddies, surface currents) formulate physical pathways for gene flow. The southern basin has lower physical connectivity than elsewhere, agreeing with known genetic structure of coral reef organisms. The central Red Sea provides key source regions, meriting conservation priority. Our analysis demonstrates a cost-effective tool to estimate biophysical connectivity remotely, supporting coastal management in data-limited regions.

## Introduction

Coral reefs occupy less than 0.2% of the world’s oceans, yet they host 35% of all known marine species^1^. These fragile ecosystems face serious threats from climate change, ocean acidification, destructive and unsustainable fishing practices, and water-polluting land-use activities^2, 3^. Metapopulation connectivity, the exchange of individuals and genes among geographically separated sub-populations, is a key process for maintaining biodiversity and resilience in coral reef ecosystems^4^. Effective descriptions of connectivity pathways hence remain a major goal in marine ecology and conservation^5, 6^.

The life-history of most marine species is characterized by a dispersive larval phase that connects relatively sedentary adult populations^4, 7^. Connectivity pathways in marine systems are hence inherently driven by an interplay of physical factors (e.g., eddies, longshore currents) and larval traits (e.g., vertical migration, larval duration)^4, 8, 9^. Ocean circulation may thus act as a vector or inhibitor of gene flow across various spatial scales^10, 11^. The integration of oceanographic models with empirical genetic data has revealed the power of physical pathways to predict spatial population structuring for a range of species^12, 13^. Tracing large-scale physical connectivity pathways would thus bring insights into population dynamics and spatio-temporal patterns of gene flow^14–16^, ultimately facilitating the effective design of marine protected area networks^17^. Circulation models of sufficient resolution and quality, however, are often lacking in remote areas of conservation concern, primarily due to the high costs of *in situ* data collection^18^ and logistical issues in politically diverse regions, e.g. the Arabian Seas^16^ and the Caribbean^11^.

Satellite-derived observations can effectively provide global views of natural processes, including surface water movement at synoptic scales not feasible through conventional field measurements^19, 20^. Remote sensing may thus provide a cost-effective approach to estimate physical pathways of larval connectivity in regions of limited data availability.

Here, we implement a multidisciplinary approach, integrating satellite-derived observations, ship-borne *in situ* profiles, particle dispersion simulations and genetic population data to visualize meso/large scale physical pathways and to investigate their role in driving gene flow among coral reef systems in the Red Sea. Reef communities (including benthic invertebrates and fishes) have been shown to exhibit remarkable genetic homogeneity across most of the Arabian Peninsula coastline, except at the southern part of the basin^21–24^. Here, we hypothesize that dynamic circulation flow could reflect strong basin-wide physical connectivity; yet, such linkages remain undemonstrated. We computed long-term (>20 yrs.) connectivity estimates of simulated particles driven by satellite observations of sea level elevation to create a measure of pairwise oceanographic distance among coastal populations. The outputs were then linked with an existing dataset of genetic distance among populations of anemonefish, *Amphiprion bicinctus*, along the eastern Red Sea coastline^15^. Our approach presents a blueprint for the utilization of satellite-derived sea surface observations to predict avenues of connectivity in marine metapopulations.

## Results

### Tracing connectivity pathways via satellites

**Figure 1:**
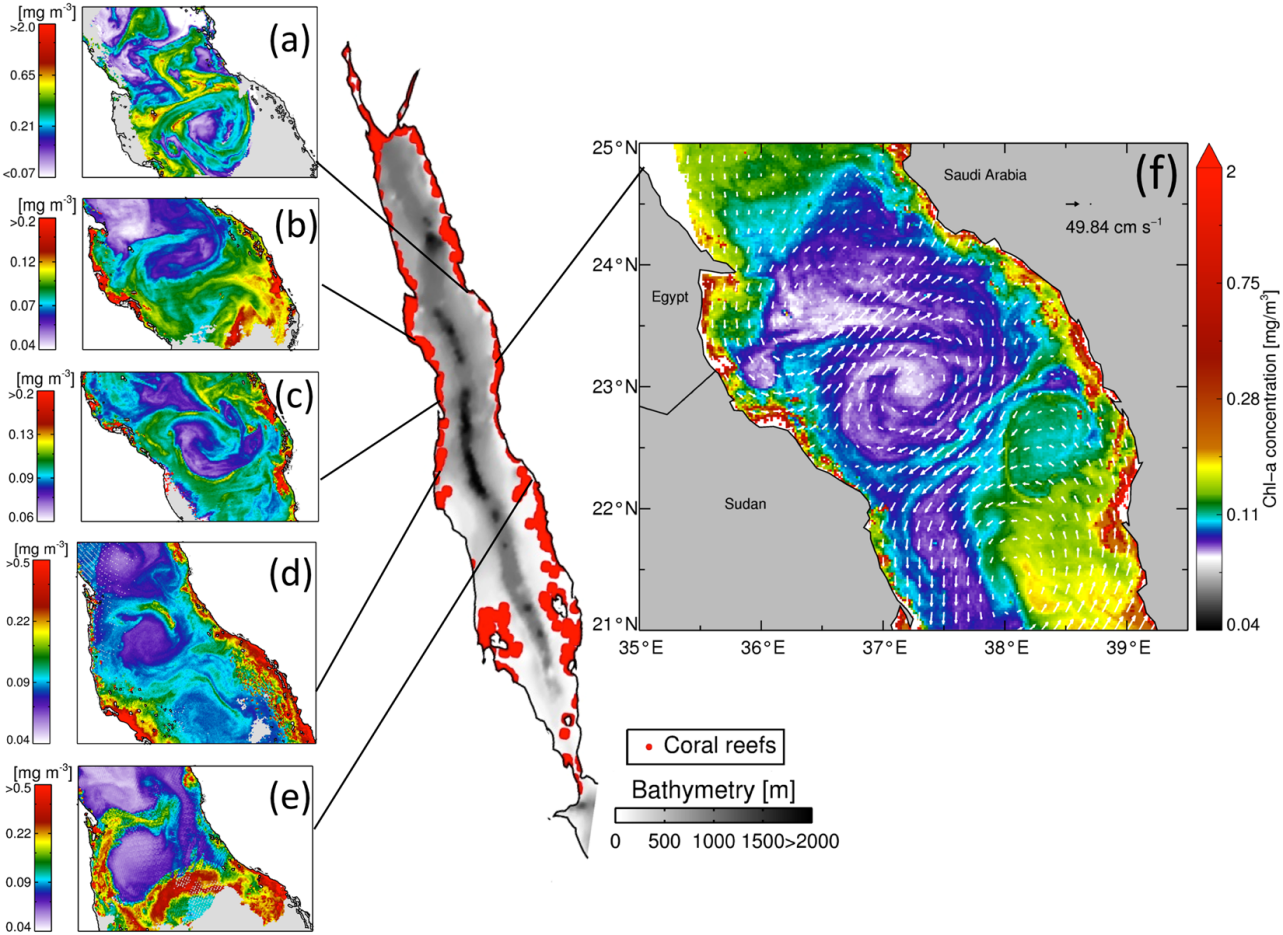
Remotely-sensed ocean colour observations portraying surface flow (eddies and currents) between coral reefs in the Red Sea. a-e, Chlorophyll (mg/m^3^) patterns during different time periods (1-Jun-2010, 21-Jun-2009, 4-Mar-2006, 13-Apr-2001, and 14–16-June-2004, respectively), and f, altimeter-derived geostrophic currents superimposed on chlorophyll observations, depicting circulation-driven water masses among coral reefs (29th March 2010). The central map depicts sea-floor elevation and the position of coral reefs (red circles) in the Red Sea (coral reef positions were acquired from: Global Distribution of Coral Reefs, 2010)^41^. (Plots created using IDL 7.1, http://www.harrisgeospatial.com/docs/using_idl_home.html).

**Figure 2:**
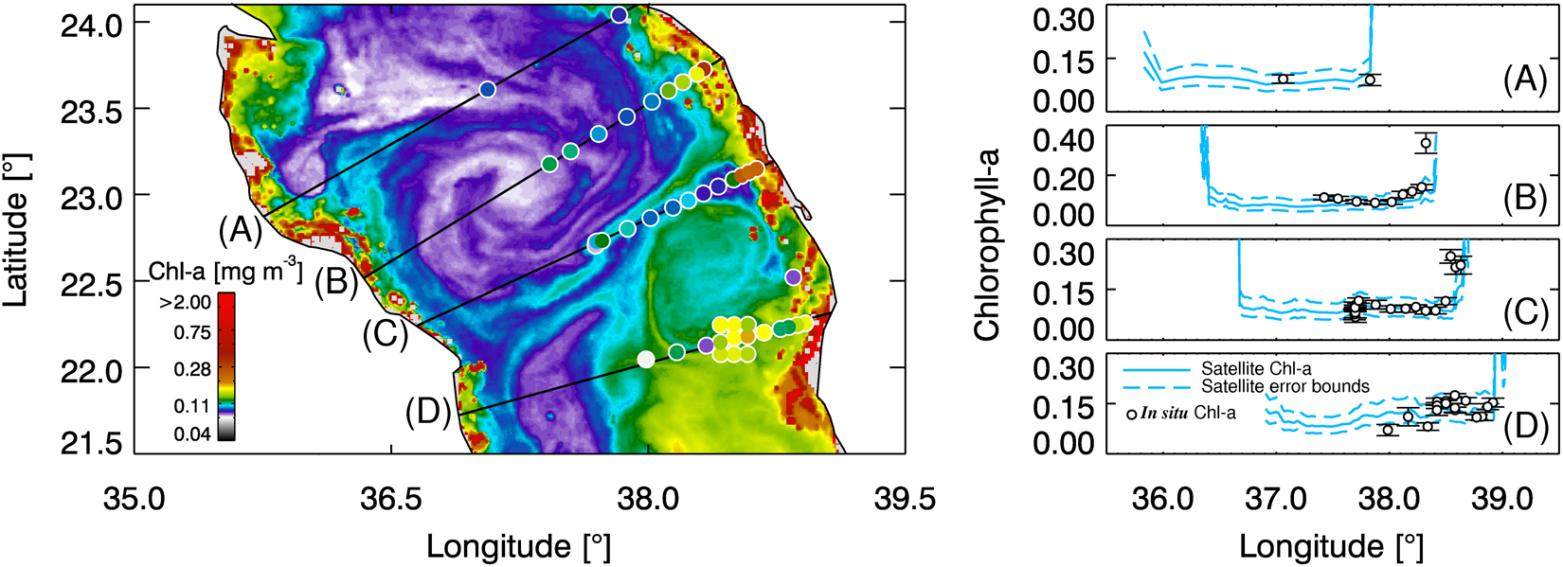
Concurrent ship-borne and remotely-sensed chlorophyll concentrations (mg/m^3^) in the central Red Sea. *In situ* concentrations (white circles) were superimposed, using the same colour scale, onto the cloud-free satellite-derived chlorophyll image acquired during the time of the cruise (mid-March 2010). (**A**–**D**) Chlorophyll concentrations (including standard error) along the transects from coastal to offshore waters. (Plots created using IDL 7.1, http://www.harrisgeospatial.com/docs/using_idl_home.html).

Sensing circulation flow (via ocean colour and geostrophic currents) remotely from space, we detected signs of water mass advection and meso-scale eddy formations along the entire Red Sea basin (Fig. 1). Water masses were dragged away from coral reef complexes and transported hundreds of kilometers away from the west coast of the Red Sea (Africa) to the east coast (Arabian Peninsula) and *vice-versa*. Occasionally, these circulation pathways were interrupted or redirected locally by smaller scale eddies and filaments (Fig. 1b,d and f). Circulation flow can be evidently inferred from the surface signature of altimeter-derived geostrophic currents. However, altimeter-derived geostrophic currents may be limited in realistically representing surface circulation in narrow seas (see Potential Bias section). It is hence important to authenticate such remotely sensed patterns. To examine if satellite-derived geostrophic currents adequately captured circulation dynamics, we overlaid concurrent altimeter observations on ocean-colour imagery (Fig. 1f). Altimetry revealed a mesoscale clockwise eddy in the central Red Sea that transported productive water masses from the east (23.3°N) to the west coast (~22.2°N), at an approximate speed of 25 cm s^−1^ (Fig. 1f). Simultaneously, slightly further south (22.7°N), an anticlockwise eddy transferred water masses of higher chlorophyll concentration the opposite direction (~21.8°N). Around 23°N, chlorophyll concentrations gradually decreased as water was transported away from the east coast into oligotrophic offshore waters. Interestingly, as the clockwise eddy continued to rotate, chlorophyll concentrations increased again as water masses converged with the west coast (22.8°N). These patterns indicate that eddy-driven, oligotrophic water masses may be (re-)fertilized while passing through coastal reefs. This enhanced productivity may be important for larvae survival^25^ (Supplementary Text 1), yet these higher chlorophyll concentration signals have not been verified due to the lack of in-water measurements. We integrated our satellite-derived observations with ship-borne measurements of chlorophyll concentrations taken along transects from coastal to offshore waters in the central Red Sea (Fig. 2). These concurrent *in situ* measurements corresponded markedly with our remotely-sensed observations, showing a steep gradient of decreasing chlorophyll concentrations with increasing distance from coastal coral reefs at approximately 39°E.

Our findings indicate consistency between altimeter-derived geostrophic currents and the surface circulation patterns from ocean colour in the Red Sea. In addition, several studies have evaluated the remotely-sensed sea surface height with different independent *in situ* observations, suggesting it adequately captures surface current and circulation patterns in the Red Sea (Supplementary Text 2). Overall, we found that surface currents appear to transfer productive water masses, connecting fringing coral reefs across and along the Red Sea basin. These results motivated an investigation into physical connectivity using altimeter-derived geostrophic currents.

### Surface circulation forms avenues of gene flow

**Figure 3:**
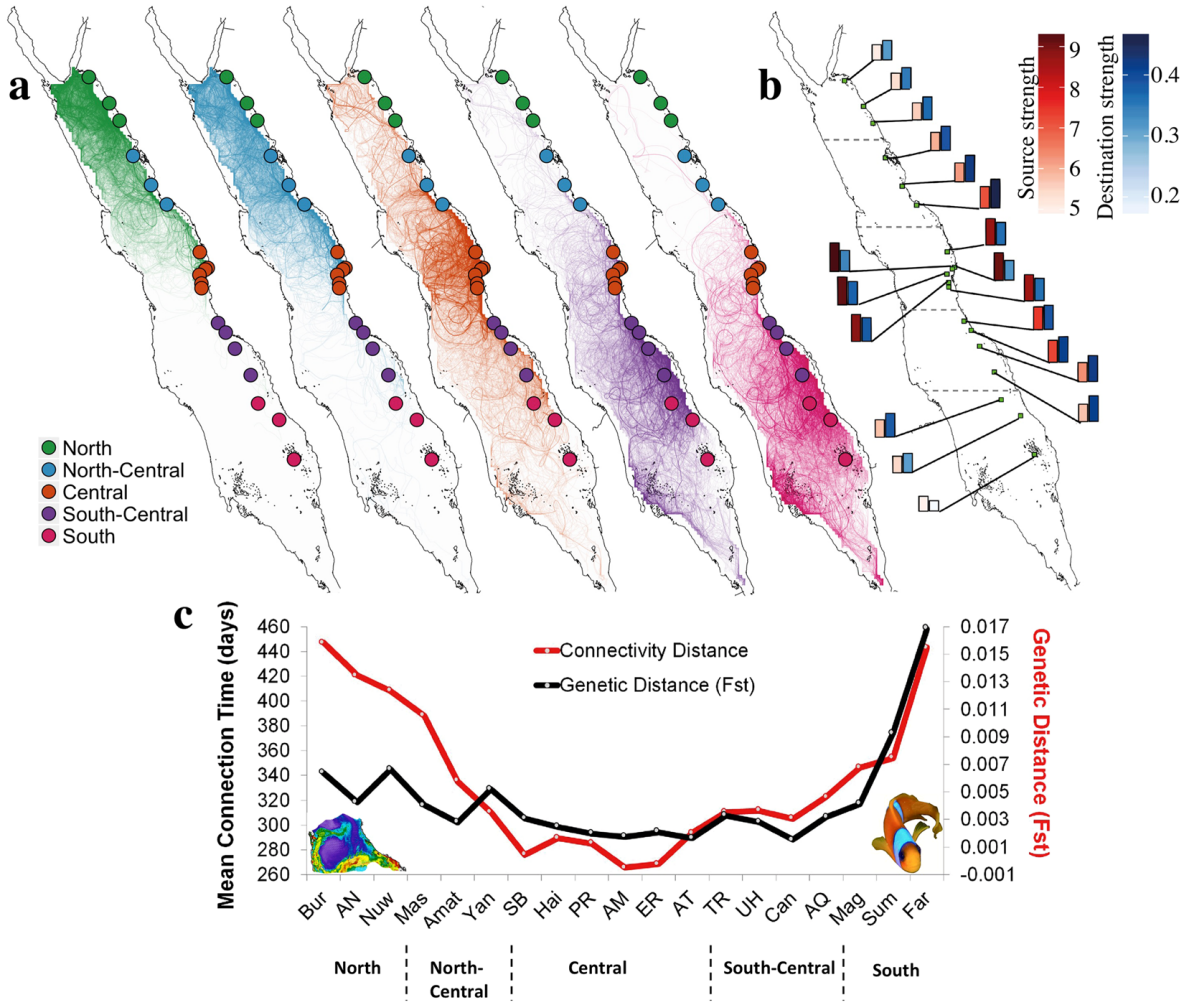
Particle dispersion trajectories (simulated using >20 yrs. of satellite-derived geostrophic velocities), forming pathways of connectivity in the Red Sea. (**a**) Particle releases at different sample locations along the east coast of the Red Sea. The different colours denote each of the five different provinces, derived by cluster analysis on pairwise Mean Connection Time –MCT (days) (Supplementary Fig. S1). (**b**) Source and destination strength for each sample site. Strength is indicated by both colour scale and histogram size. (**c**) MCT versus mean genetic distance patterns of a common reef fish (*Amphiprion bicinctus*). The particle release sites were based on the genetic sampling locations, and the exact position can be found in Supplementary Table S1. The *A. bicinctus* image was taken by Dionysios Raitsos in the Red Sea. (Plots created using IDL 7.1 software, http://www.harrisgeospatial.com/docs/using_idl_home.html).

To investigate the role of surface circulation in forming avenues of population connectivity in the Red Sea, we ran a passive particle dispersal model forced by altimeter-derived surface circulation over a 20 year period (1992–2012)^26^. We released a total of twelve million particles from 19 locations spanning approximately 1500 km of the eastern Red Sea coast (Fig. 3a). Particle trajectories indicated that the central Red Sea is connected with nearly every region of the basin, followed to a lesser degree by the near-central provinces. The southern Red Sea appears to be the least connected region (Fig. 3a; Supplementary Fig. S2). To assess source-sink dynamics across the east coast of the Red Sea, we further calculated histograms of origin and destination strengths^27^ for each of the release sites (Fig. 3b). Again, results indicated that the central Red Sea is an important source area for the rest of the basin, while the southern province holds the lowest source and destination strengths (an indication that this area is less well connected). All other sampling sites showed high destination strengths, implying generally strong connectivity between reefs along the eastern Red Sea coast.

**Figure 4:**
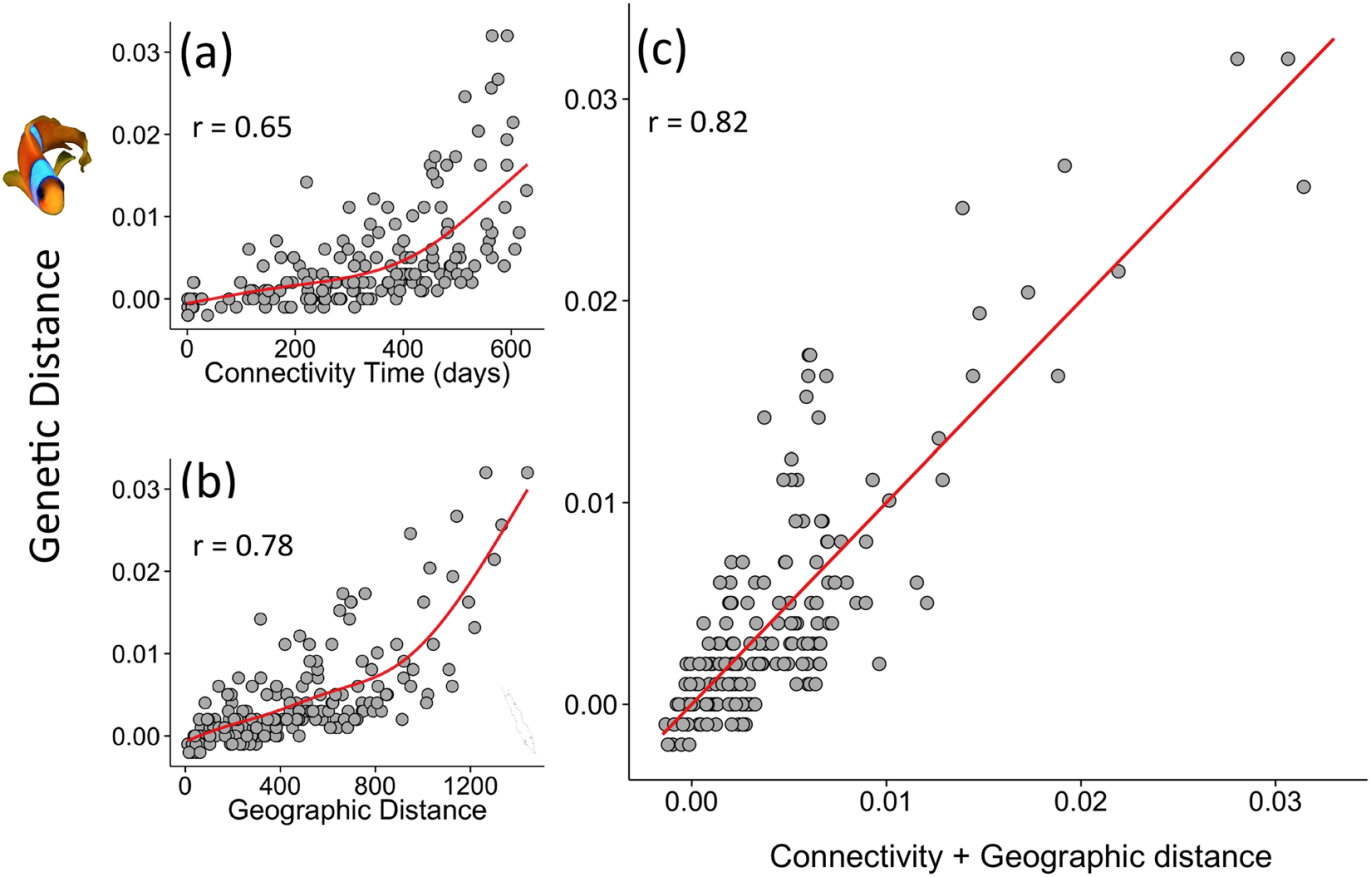
Relationships between pairwise genetic distances (anemonefish), satellite-derived circulation flow and geographic distances along the east coast of the Red Sea. The scatterplots show the relationships between: (**a**) gene flow and remotely-sensed circulation flow distances (IBF, days), (**b**) gene flow and geographic distances (IBD, km), and (**c**) the combined effects of circulation flow and geographic distances on genetic distance. The analysis was performed with Multiple matrix regression with randomization (MMRR), fitted with a Generalised Additive Model (GAM) regression.

To evaluate the potential effect of oceanographic forcing on pathways of gene flow in the Red Sea, we compared results from our passive dispersal model with an existing genetic dataset generated from *A. bicinctus* along the east coast of the Red Sea^15^. Based on these particle tracking simulations, we calculated a distance matrix of Mean Connection Time (MCT in days) between release locations^27^. Briefly, MCT is defined as the average time for a passive particle released at site (i) to reach site (j), with low MCT values indicating high connectivity, and *vice-versa* (see methods). Results showed that mean patterns of genetic distance (pairwise F_ST_) among sampling sites paralleled the overall MCTs among release sites, indicating that genetic differentiation between populations are linked to physical oceanographic pathways (Fig. 3c). To further quantify these relationships, we used the MCT matrix to assess linkages between geographic, genetic and oceanographic distance using models of isolation-by-distance (IBD) and isolation-by-circulation (IBC), fitted with a Generalised Additive Model (GAM)^28^. We found highly significant relationships for both the IBC (r = 0.65, p < 0.001) and IBD model (r = 0.78, p < 0.001) (Fig. 4a,b). A combined model of both predictors (circulation and geographic distance) further improved the fit significantly (r = 0.82, p < 0.001) (Fig. 4c). The choice of the final model was based on Akaike information criterion (AIC), which compared the significance and the performance of every model (e.g. each predictor separately or combined) to assess the best model fit in explaining genetic structure. The latter results suggest that dynamic circulation features (eddies and surface currents) may formulate physical pathways for gene flow in the Red Sea.

**Figure 5:**
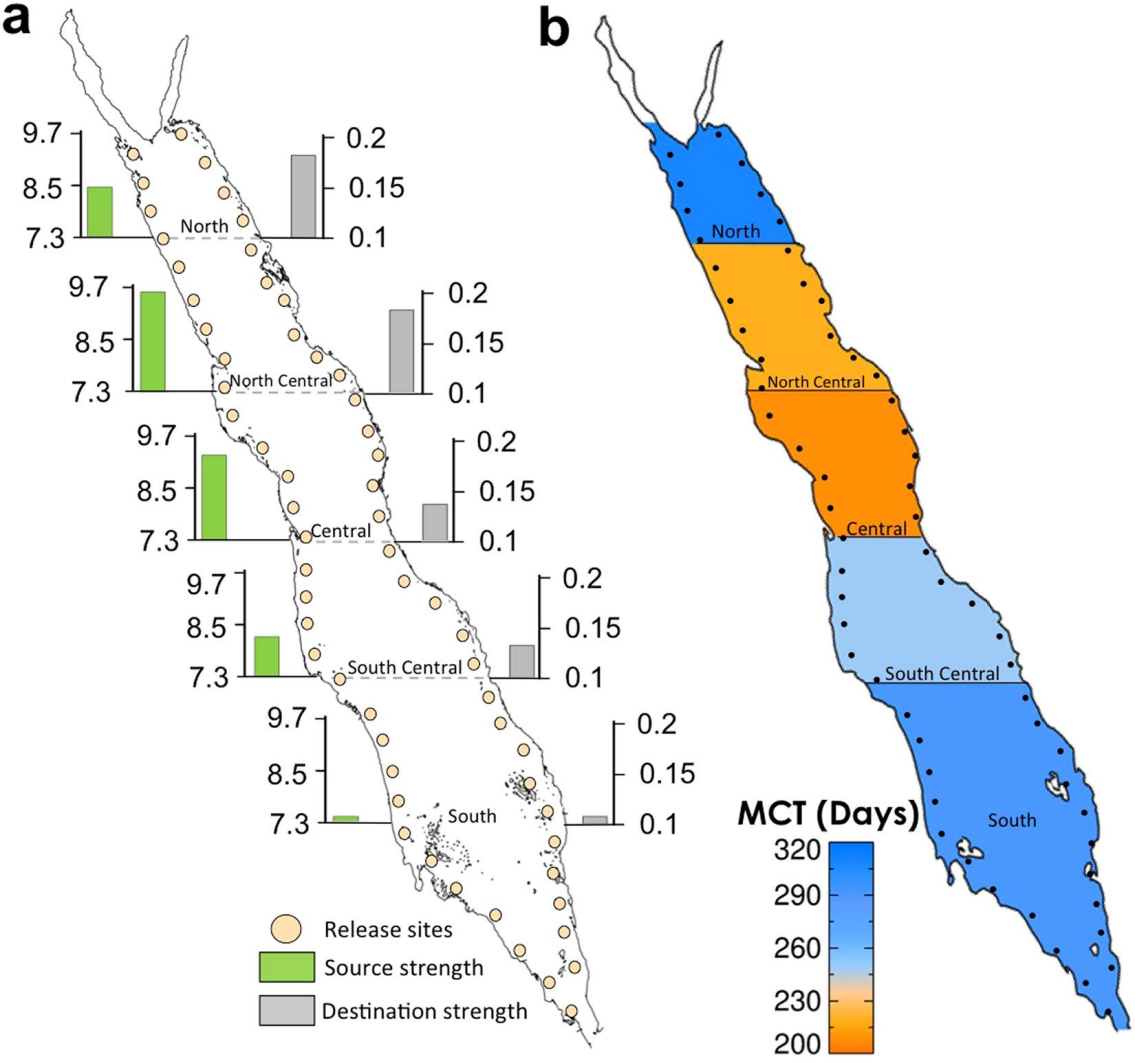
Average source-sink dynamics and Mean Connection Time (MCT) based on 60 sites along the east and west coasts of the Red Sea. (**a**) Source and destination strength averaged for each of the five different provinces (derived by cluster analysis, Fig. S1). Histograms depict the strength of the dynamics (source strength in green colour and destination in gray). (**b**) Mean Connection Time (days) averaged for each province. The different colours denote the average time taken for particles from one province to reach the rest provinces. The central Red Sea province requires the minimum amount of time (MCT) to connect to the rest of the provinces. (Plots created using IDL 7.1 software, http://www.harrisgeospatial.com/docs/using_idl_home.html).

It has to be acknowledged that the estimated network metrics (MCT and source/destination strength) were based on the 19 genetic-sampling regions only. Regardless of how well the pre-selected sites spread along the east side, they do not necessarily represent basin-scale patterns of coral reef networks. To address this issue, as well as to gain further confidence in the aforementioned outcomes, we repeated the experiment by releasing particles at 60 sites (spread at equal latitudinal distances), covering the majority of the Red Sea coral reef complexes (Fig. 5). Overall source-sink dynamics and MCT were then calculated for each province. The analysis over the whole basin confirmed our previous findings: the central provinces (central/north-central) displayed the highest source strength, while the southern province exhibited the weakest source-sink dynamics in the Red Sea (Fig. 5a). Similarly, the central province required the least amount of time (MCT) to connect to the rest of the provinces, while the north and south ends required the longest connection times (Fig. 5b). In conclusion, the results confirm that the central Red Sea is an important source region, while the southern end is relatively isolated in relation to the rest of the basin.

## Discussion

Unequivocally, a deeper understanding of the key ecological processes of marine larval dispersal and recruitment requires a better comprehension of the physical pathways of connectivity^4, 19, 29^. Biophysical dispersal models can simulate patterns of connectivity in coral reef ecosystems; yet, in many tropical regions uncertainties arise due to inadequate oceanographic knowledge and data. Our analysis highlights the successful use of satellite-derived surface currents in dispersal models. The geostrophic currents effectively predicted patterns of spatial genetic variation in a species with pelagic larval dispersal. Our results reveal that the surface water circulation of the Red Sea is a key factor in regulating patterns of gene flow among distant coral reef communities.

Large coral reef complexes occupy nearly the entire coastal zone (>5000 km) of the Red Sea. Previous studies have demonstrated a surprisingly homogeneous community structure^24^ as well as marked genetic homogeneity across a range of coral reef taxa (including a species of fish^15^, coral^23^ and sponge^21^) along most of the east Red Sea coast (>1500 km), excluding the southern end. Overall, our analysis showed that the dynamic surface circulation may play a key role in these genetic population patterns. Geographic distance was also a significant explanatory variable of genetic structure. We acknowledge that the genetic data used here were irregularly distributed along the Arabian coastline (i.e., the central Red Sea comprising a substantially higher number of sampling locations in comparison to the rest of the provinces), hence physical proximity may have increased significance of the IBD relationship (Fig. 4b). This aggregation of the sampling sites in the central Red Sea could reduce our confidence that the central provinces are indeed key source regions (Fig. 3b). To account for this issue, we ran an additional experiment, releasing particles at equally-spaced sites covering the entire Red Sea. The results further corroborated the essential role of the central provinces as key source regions, which also required the least time (MCT) to connect with the rest of the basin (Fig. 5). Based on our analysis we advocate prioritization of conservation initiatives in the central part of the basin.

The central part of the basin exhibits the highest frequency of eddies; these circulation features have the largest mean zonal radius and the most prolonged lifespan (6–9 weeks) in comparison to the rest of the basin^26^. In contrast, the southern end of the Red Sea (e.g. Farasan Islands) exhibited the lowest source/sink strength, indicating poor connectivity with distant reefs in the Red Sea. The described patterns concur markedly with recent genetic research in the region, demonstrating homogeneity in population structure along the east Red Sea coast that is interrupted by a sudden genetic break at around ~19°N in two different taxa^15, 21, 30^. The reduced circulation activity^26^, and the low source/sink connectivity dynamics in the southern region, may not be the only reasons for the genetic heterogeneity between the southern end and the rest of the basin. The regional circulation dynamics of the southern Red Sea (water influx from the Indian Ocean) could also partly explain the heterogeneity in genetic population (Supplementary Text 3). While the aforementioned studies^15, 21, 30^ ascribed the genetic break to a potential phenotype-environment mismatch for recruits passing across a eutrophic-oligotrophic transition^31–33^, our results indicate that physical forcing plays a key role in explaining the observed genetic patterns.

Circulation patterns in the Red Sea have previously been characterized as highly dynamic^14, 26, 34–36^, facilitating water mass exchange among coral reefs by mesoscale features. We show water mass movement from coastal coral reefs to offshore waters and towards distant coastal regions. Besides mere transport, these productive water masses may be important for larval survival. Increased productivity and encounters with mesoscale eddies have been shown to enhance survival of pelagic larvae^4, 25, 37, 38^. These survival advantages may carry over beyond transitions into oligotrophic waters^39^ and potentially beyond settlement^38^ (see supplementary Text 1).

The speed of water mass transport is another important factor, as larvae have to reach their destination within a species-specific window of time (i.e., before energy reserves are depleted or before metamorphosis)^18^. Here, we observed water masses, trapped in meso/large scale circulation features, traveling among distant reefs (>250 km away) in less than two weeks, theoretically facilitating successful dispersal for the majority of coral reef species^11^. The circulation speed reported here is largely coherent with previous studies^26^, but even faster coastal currents (30 cm s^−1^) have been observed in the Red Sea^40^. Maximum reported eddy velocities have been shown to reach up to 100 cm s^−1^ ^14, 35^.

Coral reefs are among the most biologically diverse ecosystems on Earth. This biodiversity translates directly into food security and societal support through fisheries, recreation, and tourism. Tracing connectivity patterns could improve the design of marine reserve networks in vulnerable biodiversity hot-spots. Sensors onboard satellite platforms continuously sample the Earth at synoptic scales, providing long-term global observations of the oceanic surface, thereby providing a cost-effective tool to detect and model coral reef connectivity. By sensing major avenues of connectivity from space, we provide a baseline towards effective management of a fragile ecosystem, the Red Sea. Our approach could be applied to any region where shortages of detailed oceanographic information are hampering coastal management. Present day precautionary conservation measures and management practices may have a profound influence on the future functioning of coral reef ecosystems, and consequently for society.

## Methods

### Datasets

#### Satellite Remote Sensing and other datasets

Remotely-sensed Geostrophic currents: Geostrophic velocities were used in the numerical simulation of particle dispersion (see below) to calculate connectivity between the coral reefs. To compute the geostrophic velocities, the Absolute Dynamic Topography (ADT) data, the latest version of the SSALTO/DUACS products distributed by AVISO based on multi-mission satellite altimeter observations, were acquired from ftp://ftp.aviso.oceanobs.com/global/dt/upd/msla/merged/. Daily data were mapped with a Cartesian 1/4 degree grid and geostrophic velocities were computed from ADT. A detailed methodological approach on satellite-derived geostrophic currents and its application in the Red Sea can be found in Zhan *et al*.^26^.

To plot the bathymetry in the Red Sea (Fig. 1), we obtained ETOPO5 sea-floor elevation data on a 5-minute latitude/longitude grid from the National Oceanographic and Atmospheric Administration (NOAA) at: http://www.ngdc.noaa.gov/mgg/global/ etopo5.HTML.

Coral Reef positions: The Global Distribution of Coral Reefs dataset (version 2010) compiled by the UNEP-WCMC was used to locate the position of coral reefs in the Red Sea (Fig. 1). The dataset sources include the Millennium Coral Reef Mapping Project (IMaRS-USF (Institute for Marine Remote Sensing- University of South Florida) & IRD (Institut de Recherche pour le Developpement, 2005) and the World Atlas of Coral Reefs^41^.

Ocean Colour datasets: Acker *et al*.^16^ demonstrated the value of using remotely-sensed ocean colour to trace surface water movement in the Red Sea, showing a mesoscale anticyclonic eddy transferring waters of elevated chlorophyll content from the African coastline into offshore regions. Here, we processed ocean colour datasets to visually detect surface circulation patterns, but also clearly show that these circulation features transfer productive water masses between distant coral reefs in the Red Sea. We used daily, 1 km resolution, Level-2 remotely-sensed surface chlorophyll (mg/m^3^) measurements from the Moderate Resolution Imaging Spectroradiometer (MODIS-Aqua) and the Sea-Viewing Wide Field-of-View Sensor (SeaWiFS) produced by the NASA Ocean Biology Processing Group, and available from the NASA website (http://oceancolor.gsfc.nasa.gov/). All figures were produced using regionally-tuned chlorophyll algorithms, designed and validated for the Red Sea^42, 43^. The aim of this study was to use ocean colour signals as a tracer of spatial variations, rather than an estimate of absolute chlorophyll values (see Potential Bias section). The example images were selected based on several criteria, such as cloud-free composites, connecting disparate coral reefs across the two continents, depicting coastal currents, cyclonic and anti-cyclonic features, and focusing on the oligotrophic spring and summer months (when the enhanced productivity could be particularly important for larval survival^16, 25, 31, 44^). The ocean colour images were derived from MODIS (Fig. 1a,b,c,e,f) and SeaWiFS (Fig. 1d). Level-2 quality control flags were turned off to show the circulation features and maximize coverage.

Chlorophyll in-water measurements: We used these datasets to validate the satellite-derived chlorophyll over coral reefs, and show that surface currents transfer productive water masses between distant coral reefs across and along the Red Sea basin. The chlorophyll data were collected during a research cruise as part of the Research Cruises Expedition Programme of the Red Sea Research Center at KAUST. In March 2010, the R/V “Aegaeo” sampled a substantial part of the Red Sea (from 17°N to 28°N), sampling continuous fluorescence vertical profiles at several stations using a WET Labs ECO-FLNTUs (FLNTURTD-964) fluorometer attached to a CTD. The fluorometer was laboratory calibrated prior to each cruise. For each *in situ* chlorophyll profile, the approximate depth to which satellite signals are likely to penetrate was estimated by: (i) calculating the euphotic depth from the surface chlorophyll concentration of each profile (top five measurements of the chlorophyll profile, representing ~5 m depth)^45^; and (ii) dividing the euphotic depth by 4.6 to approximate the 1st optical depth, which was assumed to be the average penetration depth of the satellite signal over the spectral range. For further details see Brewin *et al*.^42^.

Genetic dataset**:** We used an existing genetic population dataset of the anemonefish *Amphiprion bicinctus* (Rüppel 1830), comprising a total of 991 samples collected between October 2009 and August 2011 from 19 locations along 1500 km of the Saudi Arabian coastline of the Red Sea. We used these dataset to link the satellite-derived patterns of surface circulation with patterns of spatial genetic variation in a species with pelagic larval dispersal. All samples were genotyped at 38 microsatellite loci and data were analysed according to Nanninga *et al*.^15^. We used a matrix of pairwise genetic distance (linearized F_ST_) between sampling locations.

### Analysis

#### Numerical simulation of particle dispersion

To investigate particle patterns in the Red Sea (Fig. 3), the particulate trajectories were simulated using geostrophic velocity derived from remotely-sensed ADT. Particles were released from 10-km boxes centered around each of the 19 sites along the Arabian Peninsula coast of the Red Sea (release sites were chosen to coincide with genetic sampling sites in Nanninga *et al*.)^15^. We released a particle every 5 days during the whole period of available geostrophic currents (1992–2012), and monitored distribution patterns of each individual particle for a period of 2 years. Each simulation was integrated for 2 years based on daily velocity fields. The choice of the 2-year timeframe was a compromise between investigating a manageable number of particles and obtaining a meaningful number of connections between distant sites, offering a sensible representation of the average surface circulation in the Red Sea. Moreover, by releasing particles every 5 days during this 20 year period, our analysis adequately captured variability within the circulation regime. In total, ~650,000 particles were released from each site, totaling ~12 million particles. The simulation was conducted using the Connectivity Modeling System (CMS)^46^, which is a multi-scale probabilistic model of particle dispersal based on a stochastic Lagrangian framework. A land mask boundary condition was used to account for the convoluted coastline and less accurate remotely-sensed data in the offshore area. Further details can be found in Paris *et al*.^46^.

We repeated the same experiment as above with an expanded set of release sites in order to cover the full potential network in the Red Sea. In total, we released particles at 60 different sites along the west and east coast (Fig. 5). To reduce the computational effort needed for this experiment, while acquiring a manageable number of connections, we released 100 particles every month at each of the 60 sites, totaling 1.44 million particles over the whole 20-year period.

#### Ocean colour patterns

We presented several examples of ocean colour patterns to visually detect water mass advection and meso-scale eddy formations along the Red Sea basin (Fig. 1). To corroborate our results, we also used the ocean colour dataset to visually compare and authenticate the patterns of the satellite-derived geostrophic currents, which were then used to model connectivity. Finally, using *in situ* chlorophyll data, we provided evidence that geostrophic currents carry productive water masses between distant coral reefs, which may be important for larvae survival^25^ (Supplementary Text 1).

#### Pairwise Mean Connection Time (MCT) matrix

To calculate the MCT matrix between the release sites, we followed the method proposed by Mitarai *et al*.^27^. For each of the particles released from a site (i), we recorded the time of the first connection taken to reach site (j). A connection was counted once a particle reached the radius (<0.5°) within the domain of the site (j). The MCT between two sites, (i) and (j), was calculated by averaging the time of first connections within a period of 2 years. As a result, the MCT represents the overall connectivity patterns during the particle release period (1992–2012); it is independent of distance (km), integration time, and the number and intensity of circulation features. For instance, a passive particle might need 230 days to travel from site PR (located in a highly dynamic area in the central Red Sea) to site CAN (south-central), whereas it may take 368 days to travel the opposite way (Supplementary Fig. S2). To obtain a single pairwise MCT value, we symmetrised the MCT matrix by taking the minimum MCT value between site (i) to (j) and *vice versa* ^27^ (Fig. 4a,c). This consistent method for computing physical connectivity represents an overall long-term average of circulation flow between coral reefs, thus representing a robust measure to relate to genetic patterns. This approach allows for an integrated assessment of the level of connectedness between coral reef sites. In other words, our analysis depicts the strength of typical pathways of connections between distant reefs as defined by the long-term (20 years) circulation patterns.

#### Statistical analysis: adapting a non-linear approach to MMRR

To compute the Mantel and the Multiple Matrix Regression with Randomizations (MMRR), we followed the approach of Wang^47^. We adapted both tests by replacing the correlation coefficient of determination (r) computed with a linear regression between distance matrices with the coefficient computed with Generalised Additive Model (GAM)^28^ regression, to model the non-linearity between circulation distances and genetics (see Fig. 4). GAM is a flexible regression technique that models nonlinear relationships between variables using non-parametric smoothers. The smoothing functions were determined from a thin-plate basis, the dimension of which was chosen from Wood^48^. To account for spatial autocorrelation, *p* values were calculated based on an adapted version of the Mantel test^47^.

#### Source Strength and Destination Strength

Based on Mitarai *et al*.^27^, we calculated the source and destination strength for each of the 19 sites. The source strength represents the capacity of a site to transport particles to other sites. The source strength of a site (i) is defined as the average number of connections of site (i) with other sites. The higher the number, the higher the likelihood of connections of a specific site with others (via physical circulation pathways). In turn, the destination strength of site (i) is defined as the fraction of particles released at other sites that reach site (i). This analysis allows for the description of the main source-sink dynamics between the simulated populations.

### Cluster Analysis

A hierarchical clustering method was used to identify physically interconnected sub-regions in the Red Sea (Fig. S1), based on the MCT data matrix, which was computed from satellite-derived geostrophic currents (Fig. S2). At the algorithm initialization phase, each location was assigned to its own cluster. Then, the clusters were merged step by step (agglomerative approach) until only one cluster containing all locations was obtained. At each step, the two clusters to be merged were chosen such that the increase in inner variability (the sum of the squared MCT distances) of the resulting cluster was minimal (Ward’s minimum variance criterion). The hierarchical clustering results were presented as a tree, in which the height between divisions corresponds to the increase in inner variability at each agglomeration (Fig. S1). From this tree, we clustered the sampling sites into 5 eco-regions, which are defined by the geostrophic circulation of the Red Sea as seen from space.

### Potential biases

There are limitations in altimeter-derived geostrophic currents in near-shore coastal regions (see Fig. 3a, gaps in the near-shore regions). However, this issue is not affecting particle passage, as particles were re-inserted into the sea if they reached the coastline, thus ensuring their continuation. In addition, the altimeter-derived product used in this study has been successfully used to trace surface circulation and calculate eddy statistics in the Red Sea^26, 49^. Regardless, Fig. 1f shows clearly that the patterns of satellite-derived geostrophic currents coincide with that of ocean colour, thus qualitatively substantiating the observed patterns. In the Red Sea basin, the contribution of the Rossby number is negligible, facilitating adequate estimations of circulation and eddy dynamics based on geostrophic velocities (derived from SLA)^26^.

Moreover, we acknowledge the difficulty of comparing genetic population differentiation, potentially encompassing hundreds of years of gene flow, with contemporary circulation patterns. We used the longest (1992–2012) and most spatially extensive dataset available. MCT was derived from this 20-year climatological mean and every particle was tracked for 2 years after the release from each site, offering an adequate representation of the average surface circulation in the Red Sea. We are hence confident that the physical pathways used in this study constitute a suitable representation of surface flow (see Supplementary Text 2).

Finally, there are limitations in remotely-sensed ocean-colour observations in shallow waters. For instance, in optically-complex waters such as coral reef regions, yellow substances, particulate matter and coloured dissolved organic matter do not covary in a predictable manner with chlorophyll values^50^. Dynamic circulation and regional winds could trigger sediment resuspension in Red Sea coral reef complexes^16^, potentially affecting algorithm performance. On the other hand, such processes could serve as an important source of nutrients to adjacent waters^51^. In shallow regions, the chlorophyll signal may also be obscured by the effect of seafloor reflectance. A combination of these factors can result in an overestimation of chlorophyll^16, 31^. Validation studies, however, have shown generally good agreement between satellite-derived and *in situ* chlorophyll measures in offshore and shallow reef-bound coastal waters^42–44^ (see also Fig. 2), substantiating the utility of ocean colour data to study biological activity in the Red Sea. The aim here was to use ocean-colour signals as a tracer of spatial variations, rather than an estimate of absolute chlorophyll values (see also ref. 18). We clearly show that circulation patterns as retraced by ocean-colour data observations are coherent with *in situ* chlorophyll measurements, suggesting the dispersal of productive water masses.

## Electronic supplementary material

Supplementary Information
