# Supplementary material for: Sensing coral reef connectivity pathways from space
Source: Sci Rep. 2017 Aug 24;7:9338. doi: 10.1038/s41598-017-08729-w (PMC5571014; doi:10.1038/s41598-017-08729-w)
Supplement: Supplementary file 1 — Supplementary Information [file 41598_2017_8729_MOESM1_ESM.doc]

**Sensing coral reef connectivity pathways from space**

Dionysios E. Raitsos^1,2^, Robert J.W. Brewin^1,2^, Peng Zhan^3^, Denis Dreano^3^, Yaswant Pradhan^4^, Gerrit B. Nanninga^3,5^, Ibrahim Hoteit^3^

*1 -* Plymouth Marine Laboratory (PML), Prospect Place, Plymouth, United Kingdom

*2* - National Centre for Earth Observation, PML, Prospect Place, Plymouth, United Kingdom

*3 -* King Abdullah University for Science and Technology (KAUST), Thuwal, Kingdom of Saudi Arabia

*4 -* Met Office, FitzRoy Road, Exeter, United Kingdom

*5* - University of Cambridge, Cambridge, UK

**Supplementary Information (Figures and Text)**

This part contains 1 Table, 2 supplementary Figures, and 3 supplementary Texts

**Table S1**. Coral reef sampling site names and their location in the Red Sea

| **Area Code** | **Site Name** | **Latitude** | **Longitude** |
| --- | --- | --- | --- |
| Bur | Burcan | 27°54'35.46" | 35°03'55.20" |
| AN | An | 27°08'11.64" | 35°45'03.06" |
| Nuw | Nuwayshiziyah | 26°37'26.82" | 36°05'43.14" |
| Mas | Mashabi | 25°34'56.70" | 36°32'55.08" |
| Amat | Abu Matari | 24°43'23.82" | 37°09'04.20" |
| Yan | Yanbu | 24°08'58.29" | 37°40'30.18" |
| SB | Shi’b al Bayda | 22°44'32.96" | 38°46'57.42" |
| Hai | Haitham | 22°16'28.82" | 39°02'54.47" |
| AM | Abu Madafi | 22°04'10.64" | 38°46'18.04" |
| PR | Palace Reef | 22°13'25.53" | 38°58'07.67" |
| ER | Eagle Reef | 21°48'52.72" | 38°50'15.14" |
| AT | Abu Terr | 21°40'35.24" | 38°50'28.76" |
| TR | Tawil Ral | 20°38'51.38" | 39°23'41.42" |
| UH | Um Haj | 20°22'07.48" | 39°39'02.13" |
| Can | Canyon Reef | 19°53'25.32" | 39°57'38.40" |
| AQ | AQ3 | 19°06'31.92" | 40°29'20.94" |
| Mag | Maghabiyah | 18°16'22.20" | 40°44'11.22" |
| Sum | Sumayr | 17°47'14.46" | 41°26'30.60" |
| Far | Farasan | 16°37'05.41" | 41°56'16.26" |

**Supplementary Figure S1:**

***
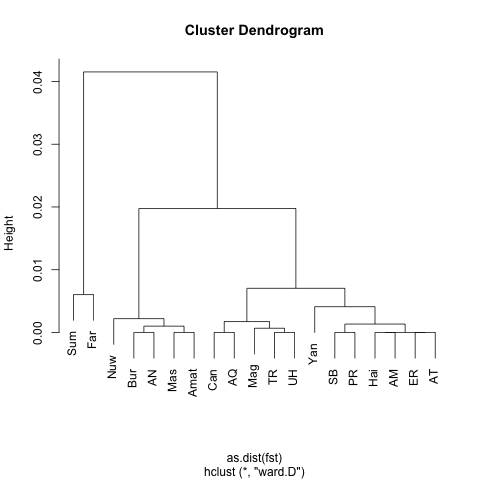
***

***Figure S1: Cluster Analysis on physical connectivity pathways derived from the 19 sites.*** Cluster analysis on the pairwise circulation flow distances divided the 19 sampling sites into 5 significant (clusters) provinces.

**Supplementary Figure S2:**

**
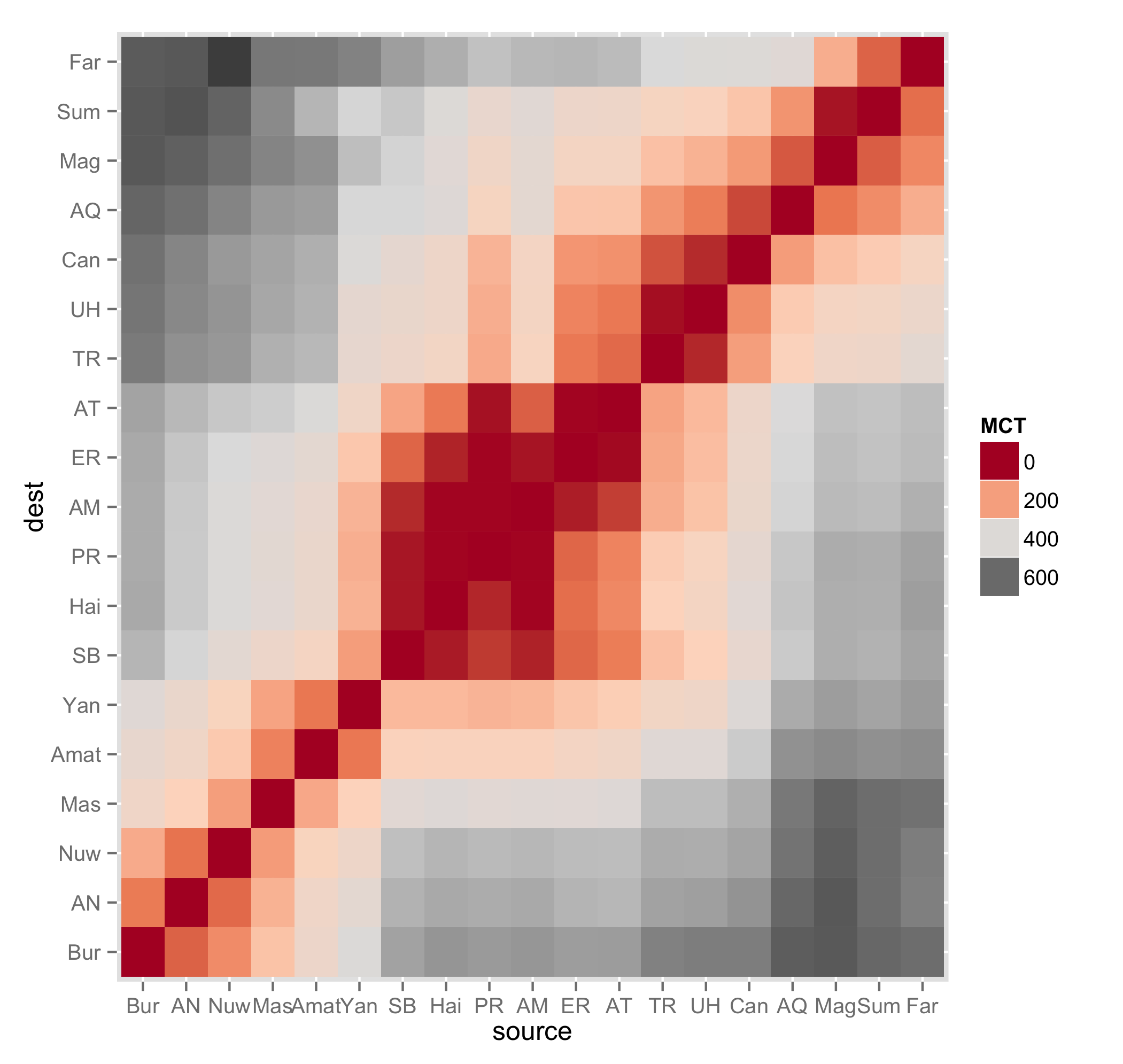
**

***Figure S2: Pairwise data Matrix on the Mean Connection Time (MCT) calculated on satellite derived geostrophic currents at the 19 sampling sites in the Red Sea.*** The connectivity relationship for each site in relation to others is depicted here. Low MCT values indicate high connectivity among the sites.

**Supplementary Text 1:**

***Importance of productive water masses***

It has been hypothesized that large coral reef complexes in the Red Sea may be sources of chlorophyll-rich detritus or nutrients, enhancing phytoplankton production adjacent to coral reefs*^16^*. Experiments conducted on the Great Barrier Reef indicate that outer reefs disseminate nutrients to open water regions*^52^*, a process that could augment phytoplankton productivity in neighboring waters*^16^*. Growing evidence shows that Red Sea coastal reefs exhibit significantly higher nutrient concentrations in comparison to oligotrophic open waters*^53,54^*. It has also been reported that nutrient export from the Gulf of Aqaba reefs (northern Red Sea) may comprise up to 50% of new production*^55^*. However, examples of such surface processes based on ocean-colour imagery have rarely been validated in the Red Sea, primarily due to the lack of *in situ* datasets*^16^*. Until now, there was no evidence that eddy-driven elevated chlorophyll values are not (partially at least) an algorithm artefact in these optically complex coastal waters (see potential biases in Methods section). Here, based on satellite-derived and ship-borne ocean colour observations, we show that coral reefs fuel higher chlorophyll concentrations to oligotrophic offshore regions and remote reef complexes. Mesoscale eddies and coastal currents trap phytoplankton-rich water masses, possibly along with other constituents from coral reefs (such as nutrients, coloured dissolved organic material, detritus etc.), covering large distances within a few weeks (Fig. 1 and 2). The latter findings are important for two reasons, (a) coral larvae undergoing development in high-nutrient areas have an improved chance of survival following transition into oligotrophic waters*^39^*, and (b) increased productivity was shown to support survival in early larval stages of corals and fishes*^4,25,37^*.

**Supplementary Text 2:**

***Quality of remotely-sensed geostrophic velocities in the Red Sea***

The altimeter geostrophic velocity (derived from gridded sea surface height) data, have been validated with different sources of independent observations in the Red Sea. Zhai and Bow^er56^ compared the surface velocity with an acoustic Doppler current profiler (ADCP) data in August-2001 (their Fig. 7 & 8). The gridded product exhibited satisfactory consistency with *in situ* observations in the southern Red Sea. Also, Chen *et al.*^57^ compared the altimeter surface velocity with several drifter data in April-2010 over the central Red Sea (their Fig. 2 & 4). Both datasets compared reasonably well with each other, depicting the strong circulation feature (eddy) in the central part of the basin. Based on these validation studies, we are confident that the satellite-derived geostrophic velocity is capable of reproducing the surface current and circulation patterns in the Red Sea.

**Supplementary Text 3:**

***Genetic population structure in relation to oceanographic processes in the Red Sea***

Circulation in the Red Sea has been characterized as highly dynamic*^14^,*^34-36,58^, with over 900 distinct individual eddy tracks identified across the entire basin*^26^*. At the same time, populations of several coral reef dwelling organisms along the Saudi Arabian Red Sea coast were shown to exhibit a genetic break at around 18-19°N, resulting in higher differentiation of the southern populations (e.g., Farasan Islands)*^15,21^*. The presence of a physical gradient at ~19**°**N has been suggested as a possible reason for restricted gene flow to the rest of the basin*^15,21,23^*. This biological differentiation is also evident in phytoplankton biomass. Reported chlorophyll-a concentrations at the Farasan Islands are five to ten times higher than those in the oligotrophic central and northern domains*^31,44^*. Calculating connectivity pathways from remote-sensing datasets, we show that the southern province of the Red Sea is the least connected area within the basin (Fig. 3 & 5). Higher chlorophyll concentrations*^31^*, low circulation activity*^26^* and low source/sink connectivity dynamics in the southern region, however, may not be the only reasons for restricted gene flow in and out of this region (physical barrier).

The southern Red Sea is unique in the sense that it is subjected to a considerable biannual influx of colder, fresher, nutrient-rich waters from the Indian Ocean via the Gulf of Aden. This influx of externally-sourced water masses into the southern Red Sea is primarily driven by monsoon winds*^3^*^6,40,58-62^. Briefly, during the winter monsoon, prevailing winds promote northward advection of colder, fresher, nutrient-rich surface waters into the Red Sea from the Gulf of Aden*^36,60,63,64^*. In addition, during summer, monsoon winds reverse their direction, causing intense upwelling in the Gulf of Aden, ultimately generating an influx of an intermediate water layer (Gulf of Aden Intermediate Water - GAIW) into the Red Sea *^33,58,61^*. The GAIW supplies the southern shallow areas and coral reef complexes with colder, fresher, nutrient-rich waters*^40^*. The northward propagation of these biochemically-rich waters (during both seasons) gradually weakens as they progress towards higher latitudes; the signal is scarcely evident beyond ~19**°**N*^33,40^,* where genetic breaks has been identified*^15,21,23^*. Recent simulated dispersion experiments in the southern Red Sea demonstrated that particles can be trapped by these water masses, and are exchanged between the southern Red Sea and the Gulf*^65^*. Fine *et al.*^66^ modeled the northward dispersal of coral planula larvae, illustrating the influx and dispersal of larvae from the Gulf of Aden (Bab-el-Mandab Straits) into the southern Red Sea (up to 18**°**N). Moreover, anemonefish specimens in the Gulf of Aden were found to be genetically similar to individuals from the Farasan Islands*^30^.* Turak *et al*.^67^ also reported that the southern Red Sea populations share more similarities with populations from the Gulf of Aden than the rest of the basin. We suggest that the southern Red Sea region may constitute an ecohydrographic gradient, rather than a pure physical one. Externally sourced water masses may form a biophysical barrier, but at the same time may also facilitate gene flow between the southern Red Sea and Indian Ocean (via the Gulf of Aden). In addition to our results (i.e., minimum physical connectivity in the southern Red Sea), the aforementioned mechanisms (water influx from the Indian Ocean) could also partly explain the distinct genetic population structure of the southern Red Sea compared to the rest of the basin.

**References:**

**52.** Szmant, A. Nutrient enrichment on coral reefs: is it a major cause of coral reef decline? *Estuaries* **25**, 743–766 (2002).

**53.** Fanning, K. A., Carder, K. L. & Betzer, P. R. Sediment resuspension by coastal waters: a potential mechanism for nutrient recycling on the oceans margins. Deep-Sea Res. 29 (8A), 953–965 (1982).

**54.** Rasheed, M., Badran, M. I., Richter, C., & Huettel, M. Effect of reef framework and bottom sediment on nutrient enrichment in a coral reef of the Gulf of Aqaba, Red Sea. *Mar. Ecol., Prog. Ser.* **239**, 277–285 (2002).

**55.** Badran, M., Rasheed, M., Manasrah, R., & Al-Najjar, T. Nutrient flux fuels the summer primary productivity in the oligotrophic water of the Gulf of Aqaba (Red Sea). *Oceanologia* **47**, 47–60 (2005).

**56.** Zhai, P., & Bower, A. The response of the Red Sea to a strong wind jet near the Tokar Gap in summer. *J. Geophys. Res.* **118**, 421-434 (2013).

**57.** Chen, C.S. *et al.* Process modeling studies of physical mechanisms of the formation of an anticyclonic eddy in the central Red Sea. *J. Geophys. Res.* **119**, 1445-1464 (2014).

**58.** Yao, F. *et al.* Seasonal overturning circulation in the Red Sea: 1. Model validation and summer circulation. *J. Geophys. Res.* **119**, 2238–2262 (2014).

**59.** Patzert, W. C. Wind-induced reversal in Red Sea circulation. *Deep-Sea Res.* **21**, 109–121 (1974).

**60.** Murray, S. P. & Johns, W. Direct observations of seasonal exchange through the Bab el Mandab strait. *Geoph. Res. Let.* **24**, 2557–2560 (1997).

**61.** Sofianos, S. S. & Johns, W. E. An Oceanic General Circulation Model (OGCM) investigation of the Red Sea circulation: 2. Three-dimensional circulation in the Red Sea. *J. Geoph. Res.* **108**, 3066 (2003).

**62.** Aiki, H., Takahashi, K. & Yamagata, T. The Red Sea outflow regulated by the Indian monsoon. *Cont. Shelf. Res.* **26**, 1448–1468 (2006).

**63.** Johns, W. E. & Sofianos, S. S. Atmospherically Forced Exchange through the Bab el Mandeb Strait. *J. Phys. Oceanogr.* **42**, 1143–1157 (2012).

**64.** Triantafyllou, G. *et al.* Exploring the Red Sea seasonal ecosystem functioning using a three dimensional biophysical model, *J. Geophys. Res.* **119**, 1791–1811 (2014).

**65.** Zhan, P. *et al.* Far-field ocean conditions and concentrate discharges modeling along the Saudi coast of the red sea. *Environ. Science Engineering*, **149**, 501-520 (2015)

**66.** Fine, M., Gildor, H. & Genin, A. A coral reef refuge in the Red Sea. *Glob. Chang. Biol.* **19**, 3640–3647 (2013).

**67.** Turak, E., Brodie, J. & DeVantier, L. Reef-building corals and coral communities of the Yemen Red Sea. *Fauna Arab.*, **23**, 1–40 (2007).
